# Normative values for circulating intestinal fatty acid binding protein and calprotectin across gestational ages

**DOI:** 10.1186/s12887-020-02142-5

**Published:** 2020-05-26

**Authors:** Darla R. Shores, Jennifer Fundora, Mitzi Go, Fauzia Shakeel, Sandra Brooks, Samuel M. Alaish, Jun Yang, Chhinder P. Sodhi, David J. Hackam, Allen Everett

**Affiliations:** 1 Department of Pediatrics, Division of Gastroenterology, Hepatology, & Nutrition, Johns Hopkins School of Medicine, 600 N. Wolfe St, CMSC 2-116, Baltimore, MD 21187 USA; 2 Department of Pediatrics, Johns Hopkins Hospital, 1800 Orleans St, Ste 8520, Baltimore, MD 21287 USA; 3 Department of Pediatrics, Division of Neonatology, Johns Hopkins Medicine All Children’s Hospital, 600 5th Street South, St. Petersburg, FL 33701 USA; 4 Department of Pediatrics, Division of Maternal Fetal and Neonatal Institute Johns Hopkins All Children’s Hospital, 601 5th Street South, St. Petersburg, FL 33701 USA; 5 Department of Surgery, Division of General Pediatric Surgery, Johns Hopkins School of Medicine, 1800 Orleans Street, Room 7337, Baltimore, MD 21287 USA; 6 Department of Pediatrics, Division of Cardiology, Johns Hopkins School of Medicine, 720 Rutland Ave, Ross 1143, Baltimore, MD 21205 USA

**Keywords:** Necrotizing enterocolitis, Biomarker, Intestinal fatty acid binding protein, Calprotectin, Neonate

## Abstract

**Background:**

Necrotizing enterocolitis (NEC) is associated with significant morbidity and mortality. Serum biomarkers to aid diagnosis, such as intestinal fatty acid binding protein (IFABP) and calprotectin, are actively being investigated; however, the normative values of these markers among healthy premature and term infants remains unknown. We sought to identify normative values for the serum concentrations of IFABP and calprotectin across gestational (GA) and post-menstrual age.

**Methods:**

We collected serum from infants (24–40 weeks GA) in the first week of life and at multiple time points in a sub-cohort of premature infants (24–29 weeks GA), excluding sepsis or known intestinal disease. IFABP and calprotectin were measured using ELISA. Groups were compared with descriptive statistics and mixed effects linear regression.

**Results:**

One hundred twelve infants had specimens in the first week of life, and 19 premature infants had longitudinal specimens. IFABP concentration in the first week of life was low and did not differ across gestational ages. Longitudinally, IFABP increased 4% per day (*P* < 0.001). Calprotectin concentration in the first week of life was more variable. An inverse relationship between day of life and calprotectin level was found in the longitudinal cohort (P < 0.001).

**Conclusions:**

Serum IFABP and calprotectin fluctuate over time. Infants had low levels of IFABP during the first week of life, independent of gestational age, and levels increased longitudinally in premature infants. Calprotectin levels generally declined over time. Normative data for infants is necessary to establish meaningful cut-off levels for clinical use.

## Background

Necrotizing enterocolitis (NEC) remains a significant challenge for clinicians caring for infants in the neonatal intensive care unit (NICU). NEC occurs in up to 7% of premature infants and has a mortality rate up to 30% [1, 2]. Survivors are at risk of intestinal failure from extensive bowel resection, as well as other complications related to severe illness and prolonged hospitalization, including lung injury and neuro-developmental delay [1, 3]. The early signs and symptoms of NEC are vague and overlap with other inflammatory processes, making NEC difficult to distinguish from more common problems in the premature infant, such as sepsis or feeding intolerance due to dysmotility. Clinicians rely on clinical signs and tests, such as changes in abdominal distention, stool output, and radiography to confirm the diagnosis of NEC [1, 4]. In many cases, the diagnosis is not made until the necrotic bowel perforates, resulting in a life-threatening surgical emergency. The development of a gold-standard diagnostic test that is highly sensitive, specific, and detects disease prior to the late findings on diagnostic imaging has been limited, in part due to the complex pathophysiology and variability in presentation of NEC. Therefore, there is great interest in identifying biomarkers that not only lead to early detection of NEC but also distinguish NEC from other etiologies of feeding intolerance.

One such promising biomarker for potential use in the diagnosis of NEC is intestinal fatty acid binding protein (IFABP), a relatively small intestinal epithelial protein involved in the transport of fatty acids found in the small intestine and colon [5]. IFABP is released into the blood after enterocyte damage. Elevations have been detected in several clinical conditions with intestinal mucosal damage, including NEC and abdominal ischemia [5–7]. IFABP is not thought to be present in the serum at high levels under normal circumstances [5]; however, normative data has not been fully established in preterm infants, as most studies have small sample sizes (20–40 control infants) [8, 9]. Recent systematic reviews have shown that IFABP appears to be more specific than sensitive in detecting NEC; however, IFABP is likely still valuable when included as part of a panel of tests [10–13]. Furthermore, determining normative data across gestational and post-menstrual ages may improve clinical utility.

Another potential biomarker for NEC and intestinal injury is calprotectin, the heterodimer S100 A8/A9 calcium-binding proteins released by neutrophils that signal activation of the innate immune system [11]. Stool calprotectin is used clinically to detect intestinal inflammation. However, stool calprotectin studies in NEC have been mixed due to highly variable normal levels in premature and term infants in the first 6 months of life as the microbiome develops [6, 14]. Serum measurements may more accurately detect a systemic inflammatory response in NEC, and the combination of calprotectin with IFABP may have value in the early diagnosis of NEC [11].

The objectives of this study were to 1) determine IFABP and calprotectin levels vary by gestational age in the first week of life, and 2) to determine if IFABP and calprotectin levels change over time in premature infants who are most at risk of developing NEC.

## Methods

We utilized blood specimens and clinical data from subjects consented (by parent/guardian) and enrolled in three prospective neonatal cohort studies approved by the Institutional Review Board.

### Subjects

Subjects were recruited from 2015 to 2018 from two tertiary children’s hospitals within the same health care system. Inclusion criteria were infants admitted to the NICU with gestational ages ranging from 24 to 40 weeks, who had blood specimens available the first week of life. We also included a sub-cohort of premature subjects with gestational ages between 24 and 29 weeks with at least 4 longitudinal blood specimens over the course of the NICU hospitalization. We excluded infants with culture-positive sepsis in the first week of life, acquired and congenital intestinal disease (such as NEC, spontaneous intestinal perforation, gastroschisis, etc.), intestinal surgery, congenital heart disease, or death prior to discharge from the NICU.

### Data collection

Clinical and demographic data were collected from the various databases. Variables included gender, race, ethnicity, gestational age, birth weight, 5 min Apgar scores, hematocrit, and chorioamnionitis (defined by clinical diagnosis or pathology findings). Type of feeding (breast milk vs. type of formula) associated with date of specimen collection was obtained.

### Biospecimens

Serum and plasma specimens were collected at various time points depending on the study in which the subject was enrolled (most subjects had daily blood specimens for the first week of life and some studies continued with weekly specimens throughout the hospitalization). For the first week of life, specimens were obtained from day of life 4–7. For longitudinal specimens, we utilized specimens obtained at approximately two-week intervals. Specimens were kept at 4 °C in the clinical lab until they were processed (typically within 48 h) into aliquot tubes and immediately frozen. Specimens were kept frozen at − 80 °C until assays were performed.

### Assays

Commercial assays were used according to manufacturer specifications for both IFABP (Hycult Biotech, #HK406–01), using a 10X dilution factor and specimen volume of 10 μl, and S100A8/S100A9 heterodimer (R&D Systems, Cat# DS8900), using a 200X dilution factor specimen volume of 1 μl.

### Statistical analysis

As this was an exploratory study, we estimated needing at least 10 subjects per two-week gestational age period (i.e. 24–25, 26–27 weeks) to see at least a 20% difference between groups. We estimated needing 15–20 preterm subjects with longitudinal specimens to see a difference over time. Our primary outcome was the biomarker level during the first week of life per gestational age group, and our secondary outcome was the change in biomarker levels over time (by day of life). We explored other clinical variables for modifying and confounding effects (gender, race, ethnicity, birth weight, and Apgar score). Sub-analyses in the subjects with available data explored the effect of hematocrit, chorioamnionitis, and a feeding type on biomarker levels.

Descriptive statistics and mixed effects linear regression equations for repeated measures with 95% confidence intervals (CI) were used to compare results between groups. Normality was determined using the Shapiro-Wilcoxin normality test. Values were log-transformed to better approximate a normal distribution, and the geometric mean of the log-transformed values was explored. To compare medians using descriptive statistics in the first week of life, differences between two groups were measured with Wilcoxin rank-sum tests, and differences between > 2 groups were compared with Kruskal-Wallis tests. Fisher’s exact tests were used to compare categorical variables. Two-tailed *P* values < 0.05 were considered significant. Statistical analyses were performed using Stata (StataCorp. 2015. Stata Statistical Software: Release 14. College Station, TX: StataCorp LP).

## Results

We enrolled 112 subjects who had specimens available the first week of life, and 19 representative premature subjects who had longitudinal specimens with 4–5 time points per subject beginning with the first week of life. See Table 1 for baseline demographics and Table 2 for birth characteristics. The following clinical variables had incomplete data: 89 subjects had hematocrit data, 62 subjects had chorioamnionitis data, and 23 subjects had feeding data.

**Table 1:** Demographics

	Total Cohort (***N*** = 112)	Longitudinal Cohort (N = 19)
**Gestational Age (weeks)**	24–40	24–29
**Race, N (%)**		
**Asian**	5 (4%)	0
**Black**	44 (39%)	9 (47%)
**White**	55 (49%)	7 (37%)
**Other**	9 (8%)	3 (16%)
**Ethnicity, N (%)**		
**Hispanic**	8 (7%)	0
**Gender, N (%)**		
**Male**	57 (51%)	11 (57%)

**Table 2:** Birth Characteristics by Gestational Age

**Total Cohort**									
**Gestational Age (weeks)**	**24–25** N = 11	**26–27** N = 18	**28–29***N* = 17	**30–31***N* = 16	**32–33** N = 12	**34–35***N* = 12	**36–37***N* = 9	**38–39***N* = 10	**40–42** N = 7
**Birth weight, g, median (IQR)**	790 (600–880)	952 (830–1060)	1170 (1090–1360)	1405 (1295–1450)	1635 (1445–2310)	2190 (2022–2665)	3008 (2660–3310)	2960 (2880–3100)	2700 (3180–3920)
**5 min APGAR, median (IQR)**	6 (4–8)	7 (4–8)	7 (5–8)	6 (5–8)	8 (8–9)	6 (5–7)	6 (4–7)	4 (2–5)	5 (3–6)
**Longitudinal Cohort**									
**Gestational Age (weeks)**	**24–25***N* = 5	**26–27** N = 7	**28–29***N* = 7						
**Birth weight, g, median (IQR)**	790 (770–860)	940 (800–1100)	1170 (880–1300)						
**Apgar score (5 min), median (IQR)**	6 (3–8)	7 (4–9)	6 (5–7)						

Abbreviations: *IQR* interquartile range

### Intestinal fatty acid binding protein

**Fig. 1:**
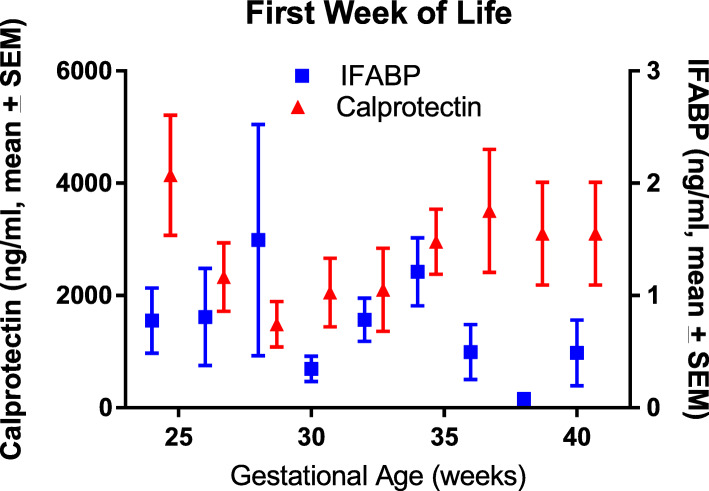
Intestinal fatty acid binding protein (IFABP, blue line) and calprotectin (red line) levels by gestational age during the first week of life (*N* = 112). For IFABP, there was a statistical difference but unclear clinical difference between gestational ages (*P* = 0.039) given overall low levels. For calprotectin, there was a significant difference between gestational ages (*P* = 0.004), but not a clear pattern

IFABP levels during the first week of life, stratified by gestational age, are displayed (Fig. 1) with means and standard errors of the mean for easiest visualization. Given the small sample size, medians and interquartile ranges were used for statistical analyses. The median IFABP level ranged from 0.01 ng/ml to 0.86 ng/dl (*P* = 0.039) per group. While there was a statistical difference, all levels were low, making a clinical difference by gestational age unlikely. There was no significant difference in median values between male and female subjects (0.013 vs. 0.171 ng/ml, respectively, *P* = 0.310). There was no significant difference in levels by race or Hispanic ethnicity (*P* = 0.077, *P* = 0.202, respectively) with most races having a median level of < 0.03 ng/ml; however, there were 5 Asian subjects in the cohort, with a median of 1.23 ng/ml. It is unclear if there is a biologic explanation or if this was coincidence given the limited number of Asian subjects in our population. The median IFABP level was the same (0.013) between those with (*N* = 18) and without (*N* = 44) chorioamnionitis (*P* = 0.239). Only 23 subjects had feeding data (7–8 per group, *nil* per os, mother’s breast milk, donor breast milk), with medians ranging from 0.32–0.62 ng/ml (*P* = 0.936). Regression models of log-transformed IFABP showed no difference in levels based on gestational age (*P* = 0.664), Apgar score (*P* = 0.170), or birthweight (*P* = 0.923). There was an association with hematocrit, with a 10% rise in IFABP per gram rise in hematocrit (*P* < 0.001); though the clinical significance is unclear as the majority (> 75%) of hematocrit levels were between 42 and 52%.

**Fig. 2:**
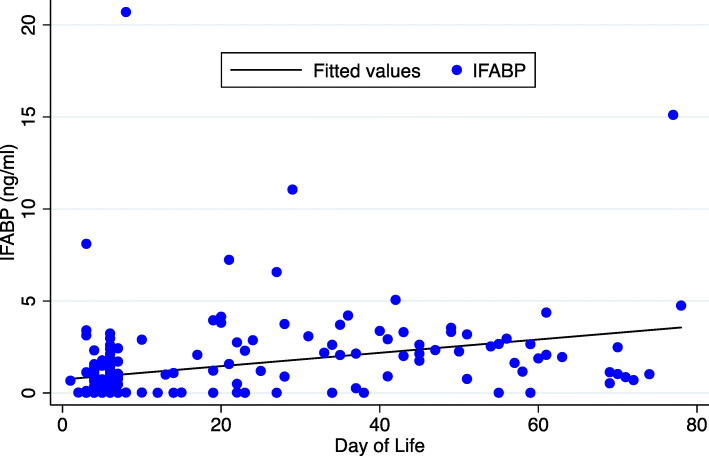
Scatterplot of repeat measures of intestinal fatty acid binding protein (IFABP) by day of life in the premature longitudinal cohort (*N* = 19) with a linear fit prediction plot for IFABP (y) on day of life (x). Levels rose by 4% with increasing day of life (*P* < 0.001), though much of the variability was not accounted for by age alone (correlation = 0.22)

Among longitudinal subjects, increasing day of life was associated with higher IFABP levels. A scatterplot of the IFABP levels by day of life is displayed in Fig. 2. The regression model comparing IFABP level with day or life showed a 4% rise in IFABP each day (P < 0.001). The median IFABP levels by day of life are displayed in Table 3. In this smaller premature cohort, race, gestational age, birth weight, Apgar score, hematocrit, and chorioamnionitis were each collinear and could not be included in the regression model. There was not an association with gender (*P* = 0.189). Nine subjects had feeding data, but most were fed mother’s or donor breast milk, and there was no difference in IFABP levels (*P* = 0.461).

**Table 3:** Serum IFABP and calprotectin by day of life (DOL) among preterm infants (24–29 weeks gestational age)

	IFABP ng/ml Median (IQR; max)	Calprotectin ng/ml Median (IQR; max)
**DOL 1–7 (*N*** **= 56)**	0.03 (0.01–1.02; 8.11)	1298 (730–2198; 10,056)
**DOL 8–14 (*N*** **= 8)**	0.51 (0.02–2.0; 20.70)	748 (596–886, 1367)
**DOL 15–21 (*N*** **= 9)**	2.07 (1.22–3.95; 7.23)	1021 (650–1329; 10,022)
**DOL 22–28 (*N*** **= 11)**	1.20 (0.03–2.89; 6.57)	784 (552–1808; 3592)
**DOL 29–35 (*N*** **= 7)**	2.62 (2.06–3.71; 11.05)	428 (345–971; 2043)
**DOL 36–42 (*N*** **= 8)**	2.53 (0.59–3.79; 5.06)	765 (588–878; 1009)
**DOL 43–49 (*N*** **= 8)**	2.48 (2.07–3.31; 3.54)	1023 (425–1639; 2528)
**DOL 50–60 (*N*** **= 12)**	2.07 (0.97–2.66; 3.19)	573 (353–917; 3321)
**DOL > 60 (*N*** **= 12)**	1.54 (0.94–3.42; 15)	608 (390–847; 2940)

### Calprotectin (S100 A8/A9)

The calprotectin level did vary by gestational age in the first week of life (*P* = 0.004), with generally higher levels in the highest gestational ages; however there were multiple outliers in the earlier gestational ages (Fig. 1). There was no difference in levels among males and females (1460 vs 1680 ng/ml, respectively; *P* = 0.803). There was no difference between ethnicities (*P* = 0.795) nor among races (1324–2074 ng/ml; *P* = 0.827). Interestingly, the highest levels were again among the 5 Asian subjects. There was no difference in levels by feeding type (*P* = 0.610). There was not a statistical difference between those with and without chorioamnionitis (2662 vs. 1298 ng/ml, *P* = 0.062). Regression models of log-transformed calprotectin showed no difference in levels based on Apgar score (*P* = 0.844) or hematocrit (*P* = 0.856). Birthweight was associated with calprotectin levels (*P* = 0.001) but was collinear with gestational age.

**Fig. 3:**
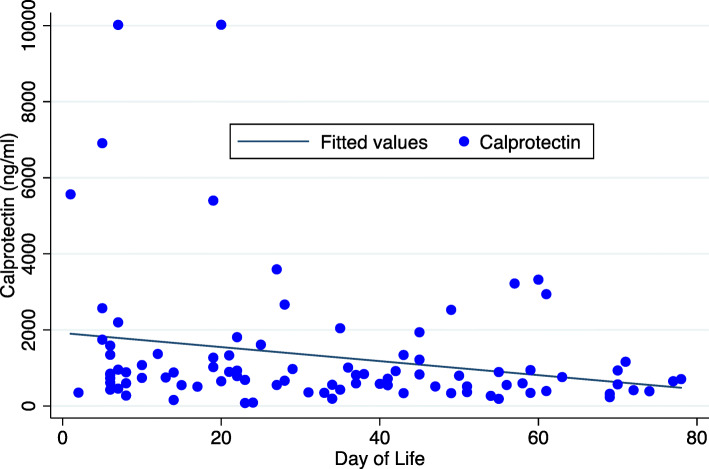
Scatterplot of repeat measures of calprotectin by day of life in the premature longitudinal cohort (*N* = 19) with a linear fit prediction plot for calprotectin (y) on day of life (x). The calprotectin level decreased by 2% per day of life (P < 0.001); however, the correlation was low (correlation = 0.13)

In the longitudinal cohort, there was an inverse relationship between day of live and calprotectin level, depicted as a scatterplot (Fig. 3). The calprotectin level decreased by 2% per day of life (*P* < 0.001). Gestational age was collinear and unable to be included in the model, as were race, chorioamnionitis, hematocrit, and Apgar scores. There was no association with gender (*P* = 0.511) or feeding type (*P* = 0.749).

## Discussion

There is an urgent need for better diagnostic tests for early detection of necrotizing enterocolitis (NEC). While IFABP and calprotectin are promising biomarkers, understanding the normal variations across gestational ages and during the early post-natal period is paramount to accurately interpreting results in a clinical setting. In a study comparing IFABP levels among premature sheep, differences were seen between extremely and moderately premature sheep, with the lowest levels in the most premature sheep [8]. Similar findings occur in other intestinal biomarkers. For example, calprotectin, a fecal marker of inflammation in the intestine, is routinely used clinically in older children and adults, but has had mixed results in the neonatal population. Very low calprotectin levels are seen in extremely premature infants, but the levels progressively increase and remain higher than normal childhood levels until after 3 months of age, even without any obvious gastrointestinal pathology [6, 14, 15]. This range of levels is in part explained by the complex interactions between with evolving intestinal microbiota and the development of immune tolerance and/or inflammation during the first few months of life.

In our study, most infants had minimally detectable IFABP levels during the first week of life, regardless of gestational age. Over 75% of subjects had an IFABP level < 1 ng/ml, with 50% having a level < 0.03 ng/ml. There was a statistical difference between IFABP levels across the gestational ages, but this is unlikely to be clinically significant as the median values were < 1 ng/ml for each gestational age group. By comparison, most proposed cut-offs for NEC have ranged from 2 to 9 ng/ml for Bell Stage 2 and 3 NEC, with even higher levels (> 19 ng/ml) reported in one study of surgical infants with a high mortality [10, 11, 13, 16]. In our cohort, there were two outliers with very high levels who did not have identifiable gastrointestinal disease.

Among the cohort of premature infants, the population most at risk of developing NEC, there were 76 repeat measures after the first week of life. Of the 76 measures, 31 (41%) were > 2.5 ng/ml and 4 (5%) were > 9 ng/ml, suggesting there would be a high rate of false-positives for the lower cut-off values. Only some of the variability in IFABP levels was explained by day of life. There was not an obvious association with demographic or birth factors, such as gender, race, birth weight, Apgar score, or chorioamnionitis. We have limited feeding data (only 50% of longitudinal subjects), with the vast majority being fed breast milk. It is possible that feeding type, and therefore intestinal microbiome, impacts the IFABP level. We also were reliant on culture results to exclude sepsis, so it is possible that culture-negative infection could have affected the results.

In contrast, calprotectin levels were more variable during the first week of life, partially explained by gestational age. The proposed cut-off level for NEC is 3000 ng/ml [11]. Twenty-nine subjects (26%) would have had false-positive results. Calprotectin is associated with sepsis [17]. While all of our subjects had negative cultures, it is possible that other etiologies of infection were contributing to the higher levels seen during the first week. After the first week of life, only 5 (7%) of the levels were above the cut-off, suggesting a lower risk for false-positive results.

There were limitations to the study, including the relatively small sample size; however, subjects were from two centers and represent the spectrum of gestational ages seen in the NICU. Furthermore, our longitudinal subjects represent the population most likely to develop NEC. Our sub-analyses were limited by missing data, particularly type of feeding. Future studies will be needed to more formally evaluate feeding type and potential associations with the microbiome.

## Conclusions

In summary, gestational age and day of life may influence biomarker levels in premature infants. IFABP was relatively low during the first week of life across all gestational ages. However, among premature infants, a substantial proportion had detectable levels with repeat measures as day of life increased, which may ultimately affect the sensitivity and specificity of IFABP for detecting NEC. Calprotectin levels were quite variable during the first week of life, which was associated with gestational age. However, levels quickly fell during subsequent measures in the preterm infants. Understanding the timing of testing in relation to post-menstrual age will be critical in interpreting results for clinical management.

## Data Availability

The datasets used and/or analyzed during the current study are available from the corresponding author on reasonable request.

## Abbreviations

CI: Confidence intervals
GA: Gestational age
IFABP: Intestinal fatty acid binding protein
IQR: Interquartile range
NEC: Necrotizing enterocolitis
NICU: Neonatal intensive care unit
